# Potential Population-Level Nutritional Impact of Replacing Whole and Reduced-Fat Milk With Low-Fat and Skim Milk Among US Children Aged 2–19 Years

**DOI:** 10.1016/j.jneb.2014.11.001

**Published:** 2015-01

**Authors:** Colin D. Rehm, Adam Drewnowski, Pablo Monsivais

**Affiliations:** 1 Center for Public Health Nutrition, School of Public Health, University of Washington, Seattle, WA; 2 United Kingdom Clinical Research Collaboration, Centre for Diet and Activity Research, Cambridge, UK

**Keywords:** energy intake, obesity, diet, policy, dairy products, child

## Abstract

**Objective:**

Dietary guidance emphasizes plain low-fat and skim milk over whole, reduced-fat, and flavored milk (milk eligible for replacement [MER]). The objective of this study was to evaluate the population-level impact of such a change on energy, macronutrient and nutrient intakes, and diet cost.

**Design:**

Cross-sectional modeling study.

**Setting:**

Data from the 2001–2002 and 2003–2004 National Health and Nutrition Examination Survey.

**Participants:**

A total of 8,112 children aged 2–19 years.

**Main Outcome Measures:**

Energy, macronutrient, and micronutrient intake before and after replacement of MER with low-fat or skim milk.

**Analysis:**

Survey-weighted linear regression models.

**Results:**

Milk eligible for replacement accounted for 46% of dairy servings. Among MER consumers, replacement with skim or low-fat milk would lead to a projected reduction in energy of 113 (95% confidence interval [CI], 107–119) and 77 (95% CI, 73–82) kcal/d and percent energy from saturated fat by an absolute value of 2.5% of total energy (95% CI, 2.4–2.6) and 1.4% (95% CI, 1.3–1.5), respectively. Replacement of MER does not change diet costs or calcium and potassium intake.

**Conclusions:**

Substitution of MER has the potential to reduce energy and total and saturated fat intake with no impact on diet costs or micronutrient density. The feasibility of such replacement has not been examined and there may be negative consequences if replacement is done with non-nutrient–rich beverages.

## Introduction

Milk and milk products are important components of a healthy diet and their consumption is recommended by numerous dietary guidelines and professional organizations.^1-3^ Milk and dairy consumption during childhood is particularly important for achieving bone health later in life.^4,5^ Milk is an important source of calcium, vitamin D, and potassium, all of which were identified as nutrients of concern by the 2010 *Dietary Guidelines for Americans* (DGA).^1,6,7^ Although children aged 2–8 years generally consume adequate amounts of calcium, adolescents aged 9–18 years fail to meet calcium recommendations and no age group comes close to meeting the threshold for adequate potassium intake.^6,8,9^

Although milk consumption has a number of benefits, in light of the obesity epidemic, concerns regarding excess energy and fat intake have emerged. National dietary guidelines and recommendations from professional organizations, including the 2005 and 2010 DGA and the American Academy of Pediatrics, recommend that children aged ≥ 2 years (and adults) consume low-fat (1% fat) and skim milk (0% fat) rather than whole (3.25% to 4%) or reduced-fat (2% fat) milk.^1-3^ Revisions to the Women, Infants, and Children standard food package finalized in March, 2014 allow whole milk for children aged < 2 years but only low-fat and skim for children aged ≥ 2 years and women.^10^ Despite these recommendations and numerous policy changes, consumption of low-fat and skim milk is low among children and adolescents. Recent data from the 2007–2008 National Health and Nutrition Examination Survey (NHANES) revealed that only 20% of children and adolescents regularly consume low-fat and skim milk, and lower-income, non-Hispanic black and Hispanic children consume the least skim and low-fat milk.^11^ Younger children (aged 2–5 years) were less likely to consume skim and low-fat milk than older children (aged 12–19 years).^11^ Driving the higher consumption of higher-fat (and also flavored) milk is a strong preference for the higher fat content of unflavored milk (and the sweetness of flavored milk).^12-14^ Because relatively few children currently consume low-fat and skim milk, evaluations should examine the maximum effect of recommendations to shift consumption from whole, reduced-fat, and flavored milk toward skim and low-fat milk, although it is unlikely that any intervention could entirely shift consumption toward the recommended milk.

The primary goal of this study was to provide quantitative information regarding the potential nutritional effects of substituting low-fat and skim milk for whole, reduced-fat, and flavored milk in the diets of children under current eating patterns. A secondary goal of this study was to determine whether such a substitution would increase diet costs, an unexplored dimension in most nutrition modeling studies. This study used data from NHANES to quantitatively examine the potential nutritional and economic impact of substituting low-fat and skim milk for whole, reduced-fat, and flavored milk in the diets of American children and adolescents from 2001 to 2004.

## Methods

### Subjects

Analyses were based on dietary intake data from children and adolescents (aged 2–19 years) who completed a valid 24-hour recall during 2 cycles of the NHANES: 2001–2002 and 2003–2004. The authors used these cycles because of the availability of food price information, which was not available for later cycles. The NHANES includes in-depth demographic, health behavior, and health outcome questionnaires, along with standardized health measurements. The National Center for Health Statistics ethics review board approved the survey and the researchers obtained informed consent. The use of this existing, publicly available dataset was exempt from human subjects review by the University of Washington Institutional Review Board.

### Dietary Assessment in NHANES

All examined survey participants were eligible to participate in the dietary interview component, which consisted of a single 24-hour dietary recall in which respondents reported all foods and beverages consumed the previous day, from midnight to midnight. The 24-hour recall data include the portion and description of each individual food and beverage consumed, based on the US Department of Agriculture food code. A set of standard measuring guides was available in the Mobile Examination Center to aid in estimating portion sizes. The NHANES staff monitored interviewers and developed criteria to determine the acceptability of each recall.^15^ Administration of the dietary recall varies by age. For children under age 6 years, the interviews were conducted by proxy; if present, the child provided supplementary information. For children aged 6–8 years, the proxy was still the primary respondent but the child was generally present and often asked to provide information. For children aged 9–11 years, the child was the primary source of information but may have been assisted by an adult who had knowledge of the child's dietary intake. Dietary recalls for children aged ≥ 12 years did not have an adult present.^15^ The examination protocol and data collection methods are fully documented elsewhere.^15^ A second 24-hour recall was completed for a subset of 2003–2004 participants but was not used here to ensure comparability of data across study cycles.

### Anthropometric Measures

To evaluate the impact of the substition models stratified by weight status, the authors used code provided by the Centers for Dieases Control to calculate BMI percentiles by age and gender.^16^ Weight status was defined as follows: underweight (< 5th percentile), healthy weight (5th to 84.9th percentile), overweight (85th to 94.9th percentile), and obese (≥ 95th percentile). Valid data for height and weight were available for 7,841 of the 8,112 study participants.

### Milk Classification

Milk was identified from the individual foods file. Whole-fat, reduced-fat, low-fat, and skim milk was identified based on the presence of these terms in the food descriptions. If these descriptors were not available, the fat content in the nutrient database was used for classification. Sweetened, flavored milk was identified as milk that contained added sugars. Cocoa and sugar mixtures, chocolate syrup with milk added and low-lactose and lactose-free milk was included in the analyses. In the case of lactose-free milk, replacement was done with non–lactose free milk, although this decision would not have altered results because the prices and nutrient values for low- and lactose-free milk were the same as for regular milk in this database. Milk shakes, smoothies, malted milk mixtures (eg, Ovaltine), soy milk, whey-based milk drinks, eggnog, buttermilk, and evaporated and condensed milk were not included in the analyses because there were not always clear lower-fat or non–sugar added alternatives to these beverages. Consumption of these items was reported 411 times compared with more than 8,500 reports of milk included in this analysis.

### Diet Cost

Diet cost estimates were based on merging the dietary recalls from NHANES with nationally representative food prices in the Food Prices Database, released by the US Department of Agriculture Center for Nutrition Policy and Promotion.^17^ In brief, diet cost, defined here as the monetary value of foods reported by each respondent, was computed from each individual's dietary recall in combination with the price database by multiplying the price per gram with the portion of each food consumed by the respondent and then summing these values for each participant. Diet cost was estimated based on all foods and beverages reported, excluding tap and bottled water. Additional details regarding the price database are available elsewhere.^17-19^

### Substitution Models

The key feature of this study was the systematic replacement of whole, reduced-fat, and flavored milk with skim and low-fat milk. The researchers used 2 substitution models to replace whole, reduced-fat, and flavored milk. The first replaced all consumption reports of this milk with white skim milk. The second used white low-fat milk. The impact of partially substituting whole, reduced-fat and flavored milk with added sugars with skim and low-fat milk was also evaluated. These models estimated the nutritional consequences of whether 15%, 25%, 50%, or 75% of children and adolescents had their consumption replaced with low-fat or skim milk. Although the complete replacement models provide information on the maximum impact of dietary changes, these partial substitution models likely represent the impact of possible interventions. Milk consumed as a standalone beverage, as well as used in recipes and with cereal, was included in all models. No lower threshold was used to identify milk consumers. An additional analysis evaluated the impact of replacing flavored milk with skim and low-fat milk on a 50–50 basis.

### Outcome Measures and Statistical Analysis

The researchers examined the population effects of substitution for all children and separately for children consuming whole, 2%, or flavored milk. Survey-weighted means were estimated for diet composition and diet cost for the observed data and the substitution models. Diet composition effects of substitution were quantified for energy (kilocalories), percent energy from total fat, percent energy from saturated fat, dietary cholesterol (milligrams), calcium (milligrams), potassium (milligrams), added sugars (teaspoon equivalents), and diet costs (dollars). Vitamin D would also be of interest, but it was not available in the nutrient database linked to these releases of NHANES. Age-, race-, income-, and weight-specific analyses for energy, percent energy from total fat, and percent energy from saturated fat were also evaluated (see Supplementary Data).

In comparing results of each of the 3 models with the observed patterns, the primary focus was on the mean differences, but the researchers also used survey-weighted linear regression models to evaluate whether the dietary changes observed were stronger than pre-specified thresholds (eg, change greater than ± 10% reference value). Reference values for cholesterol (300 mg), calcium (1,000 mg), and potassium (3,500 mg) were based on the Daily Reference Values and Reference Daily Intakes provided by the Food and Drug Administration for nutrition labels.^20^ For the percentage of energy from fat and the percentage of energy from saturated fat, 35% and 10%, respectively, were used, as identified in the 2010 DGA.^1^ The percentage of total fat was the upper-bound recommended value for children aged 4–18 years and the middle value for children aged 2–3 years. Based on the 2010 DGA, 89% of the population under study (aged 3–19 years) should consume no more than 35% of energy from fat. The reference value for added sugars was 24 teaspoon equivalents, equivalent to the mean for the population. For energy, a threshold value of 100 kilocalories was used, which corresponds to roughly a 5% change. Specifically, for all outcomes with the exception of diet cost, hypothesis testing was based on a 1-sided test because the impact of the substitution model would only result in a decrease in the dietary outcomes of interest (eg, energy or total/saturated fat). A 2-sided test was used for diet cost. All analyses used survey weights to account for the complex sampling scheme and over-sample of NHANES and were conducted using Stata 13 (StataCorp LP, College Station, TX, 2009).

## Results

### Sample Characteristics

Dietary recalls identified as valid by NHANES staff were available for 8,112 of the 8,979 respondents aged 2–19 years over both cycles.^15^ Table 1 lists sample characteristics, stratified by milk consumption.

### Milk Consumption by Age, Race/Ethnicity, and Ratio of Family Income to Poverty

Based on the MyPyramid Equivalents database, milk eligible for replacement contributed substantially to total dairy intake, accounting for 46% of total dairy servings and 69% of milk servings. Overall, 57% of children in the NHANES sample consumed whole, 2%, and/or flavored milk with added sugar. These individuals were included in the substitution modeling. Twelve percent of children consumed only skim or low-fat milk and 31% consumed no milk. Younger children (*P* < .001) were more likely to consume milk eligible for replacement (MER) than older children. Mexican American and other Hispanic children (*P* < .001) were more likely to consume MER than non-Hispanic white children. Children living in lower-income households (*P* < .001) were also more likely to consume MER than higher-income children. Significantly fewer non-Hispanic black (1.7%; *P* < 0.001) or Mexican American/other Hispanic children (6.4%; *P* < 0.001) consumed skim or low-fat milk compared with non-Hispanic white children (16.6%). Lower-income children were much less likely to have consumed skim or low-fat milk than higher-income children (*P* < .001). The small number of underweight children (prevalence, 3.7%) was more likely to consume MER than children of healthy weight (*P* < .001). Both overweight and obese children were less likely to consume MER than healthy weight children (*P* < .001 for both).

### Baseline Dietary Characteristics

Table 2 lists baseline dietary characteristics as well as the proportion above specified reference values. Mean energy intake was 2,095 and 2,142 kilocalories, respectively, among the entire child and adolescent population and consumers of MER. Average percentage of energy from total fat and saturated fat was 32.3% and 11.4%, respectively, overall and 32.7% and 12.4%, respectively, among MER consumers. Based on a single 24-hour recall, 36% of subjects consumed more than 35% of energy from total fat and 15.7% of subjects consumed more than 40% of energy from total fat. Sixty-five percent of all children consumed more than 10% of energy from saturated fat, whereas 74% of MER consumers consumed more than 10% of energy from saturated fat. Only a small proportion of children consumed too much dietary cholesterol and about half of children consumed more than 1,000 mg of calcium. Thirteen percent and 16% consumed more than 3,500 mg of potassium among all children and MER consumers, respectively.

### Substitution Effects on Energy and Nutrient Intake

The first series of substitution models replaced all whole, 2%, and flavored milk with either skim (model 1) or low-fat milk (model 2) (Table 2). For all children, replacement with skim milk resulted in a 64-kilocalorie decrease (95% confidence interval [CI], 59–69) and a 113-kilocalorie decrease (95% CI, 107–119) among children consuming whole or reduced-fat milk. Replacement with low-fat milk resulted in complementary decreases of 44 kilocalories (95% CI, 41–48) and 77 kilocalories (95% CI, 73–82) among all children and consumers of MER, respectively. Non-significant reductions in the percentage of energy from total fat were also observed among all children and MER consumers, with an absolute decrease of 3.5% (95% CI, 3.3–3.6; *P* = .32) among MER consumers in model 1. Percent energy from saturated fat was significantly reduced among children consuming MER by 2.5% (95% CI, 2.4–2.6; *P* < .001) and 1.4% (95% CI, 1.3–1.5; *P* < .001) for the skim and low-fat models, respectively. Cholesterol was reduced by 31 mg (95% CI, 29–33) and 17 mg (95% CI 16–18) for models 1 and 2, respectively, but neither change was significantly greater than the pre-specified 30-mg decrease. Very small reductions in added sugar were observed, but none of the models resulted in a significant decrease. No significant differences were observed for potassium or calcium. The effects of models 1 and 2 among specific subpopulations (age, race/ethnicity, family income, and weight status) are provided in Supplemental Data.

### Substitution Effects on Diet Cost

Diet cost was not significantly increased or decreased under model 1 (skim milk) or model 2 (low-fat milk).

### Partial Substitution Results

Table 3 includes results of substitution models that simulate the replacement of MERs to a variable degree, ranging from 15% to 75% of all consumption reports. In all models, instances of MER were replaced with low-fat and skim milk on a 50–50 basis. Changing the choices among 75% of reports would have resulted in a significant decrease of 1.4% energy from saturated fat (95% CI, 1.3–1.5; *P* < .001), but no other significant effects were observed at this intervention level or any other.

### Replacement Effects for Flavored Milk

Among the 1,159 children and adolescents (17.1%) who consumed flavored milk, replacement with skim and low-fat white milk on a 50–50 basis resulted in significant reductions in energy (132 kilocalories [95% CI, 125–139]; *P* < .001) and added sugar (4.6 teaspoons [95% CI, 4.2–5.0]; *P* < .001) (Table 4). Total fat, saturated fat, and cholesterol were marginally reduced, but this decrease was not statistically significant because most flavored milk consumed by children and adolescents were skim or low-fat. No differences were observed for calcium or potassium and diet costs were modestly but not significantly decreased.

## Discussion

The results of this study indicate that complete replacement of whole and reduced-fat plain milk with skim and low-fat milk on a per-serving basis could result in decreased energy intake and intake of saturated fat. This replacement may not affect the beneficial nutrients provided by milk, including calcium and potassium, or result in increased diet costs. As expected, the beneficial effects would be most dramatic if complete replacement were done using skim milk. However, because consumption of skim milk is low among children and adolescents, the findings for low-fat milk and those from model 2 in Table 2 may better reflect the potential effect estimates observable in the population.

The partial substitution models presented in Table 3 may provide the most useful estimate of replacement effects because many individuals are unlikely to change their milk-drinking patterns regardless of intervention. Partial substitution of MER with skim and low-fat milk resulted in significant changes in saturated fat alone, but modest non-significant effects were observed for energy and total fat. Shifting consumption from whole, reduced-fat, and flavored milk to plain low-fat and skim milk for half of US children and adolescents consuming milk would represent a remarkably successful public health intervention or campaign, but would result in only modest decreases in energy intake, total fat, and saturated fat. By comparison, the Dietary Intervention Study in Children observed a 21% increase in the number of servings of *go* dairy (eg, skim/low-fat milk, low-fat yogurt, or low-fat cottage cheese) and a 40% reduction in the number of servings of *whoa* dairy (eg, whole milk, regular cheese, and cream cheese) after 3 years of the intervention.^21^ The results presented here indicate that improving the dietary patterns of US children and adolescents may require changes to be made for multiple food and beverage groups.

It is essential to place the results of this study into the context of broader consumption patterns and trends. The effects of the first 2 replacement models are based entirely on the assumption that all whole, reduced-fat, and flavored milk is replaced with low-fat and/or skim milk. Such a replacement may not be feasible on a population level, because children may opt to consume no milk if their preferred varieties are no longer available. There is some evidence of decreases in total milk consumption and dairy nutrients after removal of flavored milk from school cafeterias, although other studies did not observe a decrease in total milk consumption after interventions sought to increase consumption of low-fat and skim milk.^22-24^ Moreover, the energy reduction resulting from a shift to lower-fat milk may be compensated for by increases in the consumption of other foods. A cross-sectional study showed that boys with lower fat and energy intake from milk generally consumed more carbohydrates, generally from nutrient-sparse foods.^25^ Because of this, the primary results (Table 2) represent an estimate of the maximal efficacy of implementing recommendations to replace whole, reduced-fat, and flavored milk with low-fat and skim milk.

Realistically, implementation of nutrition recommendations is often most feasible within institutions such as schools and child care settings. Based on additional analyses from 2003–2004 NHANES, 14.6% of milk servings from whole, reduced-fat, and flavored milk were from school cafeteria or child care settings, which indicates that emphasizing low-fat or skim milk only in those settings is unlikely to have a substantial impact on energy and macronutrient intake at the population level, because this roughly corresponds to the 15% replacement model (Table 3). Currently, policies vary concerning availability of whole, reduced-fat, and flavored milk in schools. The National School Lunch Program requires that fluid milk be either plain low-fat or plain or flavored skim milk, although this policy affects only subsidized meals.^26,27^ In addition, New York City public schools, the largest school district in the US, removed whole milk in 2006.^28,29^ Despite these and other policy interventions, author estimates based on data from 2009–2010 NHANES show that 14.1% of whole and reduced-fat milk consumed by US children and adolescents (aged 2–19 years) on Monday through Friday comes from school cafeteria or child care settings. This suggests that schools remain an important source of high-fat milk. In light of numerous efforts to shift intake away from higher-fat and flavored milk and the well-documented decreases in total fluid milk consumption observed over the past few decades, careful evaluation of milk and related nutrient intakes are warranted.^30,31^

In addition, the cost of milk, as well as other foods and beverages, has changed from 2001 to 2004, which means that estimates of diet costs associated with milk replacement may not represent the current impact of replacement on diet cost. However, although the absolute cost of milk has changed, the relative prices of skim, low-fat, reduced fat, and whole milk compared with each other have generally remained comparable. An additional limitation is the lack of data on vitamin D from these cycles of NHANES. However, because of the lack of change observed for calcium and potassium, it is unlikely that shifting consumption from whole and reduced-fat to low-fat and skim milk will have a detrimental consequence for vitamin D intake.

## Conclusions and Implications

If skim and low-fat milk were used to replace whole, reduced-fat, and flavored milk, it would be important to collect data on actual uptake of the new milk to determine whether the intervention had an unintended and perhaps deleterious impact on dietary intake (eg, students chose to opt out of milk altogether rather than consume low-fat or skim milk).^22,23,28^ In addition, if higher-fat milk were replaced with other beverages (eg, soft drinks) or no beverages, it is likely that calcium, potassium, and vitamin D intake would be reduced. Alternatively, replacement of milk with water or other non-caloric beverages would reduce calorie intake but deleteriously impact the nutrient density of diets for children and adolescents. It is important to emphasize the value of all milk and that replacement of higher-fat or sweetened milk with non–nutrient dense items could potentially reduce diet quality. Gradually shifting from whole to reduced-fat to low-fat milk may be an approach to avoid this problem.

Replacement of whole, reduced-fat, and flavored milk with low-fat and skim milk has the potential to reduce dietary energy intake and saturated fat intake. Such a replacement would have no impact on the intake of calcium or potassium and diet costs would be unchanged. However, the barriers to such a change are not clear and should be examined further before implementing interventions to shift milk intake toward low-fat and skim varieties. Programs or institutions that make an effort to replace whole, 2%, or flavored milk with skim or low-fat milk should carefully examine the uptake of low-fat and skim milk to determine whether there are any untoward consequences of intervention on the dietary habits of children and adolescents.

## Figures and Tables

**Table 1:** Consumption of Milk by Sociodemographic Characteristics Among US Children and Adolescents From the National Health and Nutrition Examination Survey, 2001–2004

	Consuming Whole Milk, 2% Milk, And/or Flavored Milk With Added Sugar		Consuming Skim or Low-Fat Milk But Not Whole Milk, 2% Milk, And/or Flavored Milk With Added Sugar		Consuming No Fluid Milk		Global *P*^a^
	n	Weighted %	n	Weighted %	n	Weighted %	
Total	4,413	56.8	623	12.2	3,076	31.0	–
Age group, y							
2–5 (reference)	1,257	73.7	111	11.1	251	15.2	< .001
6–11	1,290	63.3***	163	13.1	583	23.7***	
12–19	1,866	44.1***	349	12.1	2,242	43.8***	
Race/ethnicity							
Non-Hispanic white (reference)	1,296	55.3	383	16.6	746	28.1	< .001
Non-Hispanic black	1,374	55.4	52	1.7***	1,295	42.9***	
Mexican American/other Hispanic	1,621	64.8***	165	6.4***	944	28.7	
Other race/mixed race	122	52.5	23	8.7**	91	38.7*	
Income-to-Poverty ratio (%)							
< 130	1,960	64.1***	117	4.4	1,297	31.5	< .001
130–349	1,512	57.4**	213	11.5***	1,055	31.1	
≥ 350 (reference)	709	47.8	264	21.9***	564	30.2	
Weight status^b^							
Underweight	149	72.1**	12	5.3**	81	22.6**	< .001
Healthy weight (reference)	2,770	58.8	357	12.2	1,806	29.0	
Overweight	637	50.3**	119	14.9	499	34.9	
Obese	685	51.0***	111	11.1	615	37.9	

*.05 > *P* > .01; **.01 > *P* > .001; ***P < .001.

a *P* is from a survey-weighted global chi-square test indicating differences in milk consumption by age group, race/ethnicity, ratio of income to poverty, and body mass index status.

b Underweight is defined as < 5th percentile, healthy weight as 5th to 84.9th percentile, overweight as 85th to 94.9th percentile, and obese as ≥ 95th percentile based on Centers for Disease Control and Prevention standard growth charts.

**Table 2:** Observed and Modeled Energy, Nutrient Intake, and Diet Cost (SD) After Complete Replacement, 2001–2004

	Above Threshold (%)^a^	Observed	Model 1: Skim Milk	Model 2: Low-Fat Milk	Benchmark
Energy, kcal					
All children^b^	–	2,095 (921)	2,031 (907)	2,051 (921)	> 100-kcal decrease
MER consumers^c^	–	2,142 (929)	2,029 (908)∗	2,064 (911)	
Total fat (% energy)					
All children^b^	35.7 (47.9)	32.3 (7.7)	30.4 (8.3)	31.2 (8.0)	> 3.5% decrease^d^^,^^e^
MER consumers^c^	36.7 (48.2)	32.7 (7.2)	29.2 (8.0)	30.7 (7.5)	
Saturated fat (% energy)					
All children^b^	64.8 (47.7)	11.4 (3.5)	10.0 (3.5)∗	10.6 (3.3)	> 1% decrease^d^
MER consumers^c^	74.1 (43.8)	12.1 (3.2)	9.6 (3.3)∗	10.7 (3.1)∗	
Cholesterol, mg					
All children^b^	21.4 (41.0)	228 (193)	210 (191)	218 (192)	> 30-mg decrease^d^
MER consumers^c^	21.9 (41.3)	234 (185)	203 (182)	216 (183)	
Added sugar, teaspoons					
All children^b^	39.3 (48.8)	23.7 (17.7)	23.0 (17.6)	23.0 (17.6)	> 2.4-teaspoon decrease^d^
MER consumers^c^	37.6 (48.4)	23.3 (17.7)	22.1 (17.6)	22.1 (17.6)	
Calcium, mg					
All children^b^	42.0 (49.4)	1,002 (612)	1,027 (632)	1,012 (619)	> 100-mg increase^d^
MER consumers^c^	52.4 (49.5)	1,147 (602)	1,192 (626)	1,165 (611)	
Potassium, mg					
All children^b^	12.9 (33.5)	2,302 (1,152)	2,317 (1,160)	2,301 (1,151)	> 350-mg increase^d^
MER consumers^c^	15.9 (36.6)	2,483 (1,154)	2,509 (1,163)	2,482 (1,152)	
Diet cost (dollars)					
All children^b^	–	3.85 (1.99)	3.88 (2.00)	3.79 (1.97)	± 10%
MER consumers^c^	–	3.86 (1.92)	3.91 (1.89)	3.76 (1.89)	

MER indicates milk eligible for replacement. Note: *P* is from a 1-sided test with the exception of diet cost, which is from a 2-sided test.

∗ Difference between modeled diets and observed diets is significantly different (*P* < .001) from the specified benchmark value

a Threshold values correspond to the Recommended Daily Intake for each outcome of interest as follows: 35% energy from total fat, 10% energy from saturated fat, 300 mg for cholesterol, 1,000 mg for calcium, and 3,500 mg for potassium. There is no Recommended Daily Intake for added sugar, so 24 teaspoons was used as the threshold value

b All children refers to all children aged 2–19 years who completed a valid 24-h recall (n = 8,112)

c MER consumers refers to all children aged 2–19 years who reported consuming any type of milk eligible for replacement models, including whole-fat milk, 2% (reduced-fat) milk, and flavored milk with added sugar (n = 4,413)

d Corresponds to 10% change in reference value

e For children aged 2–3 years, the maximum percentage of energy from total fat is 40%. Among all children aged 2–3 years (n = 932), 14.8% (SD, 35.5%) consumed more than 40% of energy from total fat. Among children aged 2–3 years who were MER consumers (n = 736), 15.2% (SD, 36%) consumed more than 40% of energy from total fat.

**Table 3:** Estimated Survey-Weighted Mean Energy, Nutrient Intake, and Diet Cost (SD) After Replacing Whole and Reduced-Fat Milk With Low-Fat and Skim Milk on a Proportional Basis Among Children Consuming Whole And/or Reduced-Fat Milk, 2001–2004 (n = 4,413)

	Observed	15% Change	25% Change	50% Change	75% Change	Benchmark
Energy, kcal	2,142 (929)	2,128 (928)	2,116 (925)	2,094 (922)	2,070 (918)	100 kcal
Total fat (% energy)	32.7 (7.2)	32.3 (7.2)	32.0 (7.3)	31.3 (7.5)	30.7 (7.6)	± 3.5% change^a^
Saturated fat (% energy)	12.1 (3.2)	11.8 (3.2)	11.6 (3.2)	11.2 (3.3)	10.7 (3.3)∗	± 1% change^a^
Cholesterol, mg	234 (185)	230 (185)	227 (185)	221 (184)	215 (183)	± 30-mg change^a^
Added sugar, teaspoons	23.3 (17.7)	23.1 (17.7)	22.9 (17.7)	22.7 (17.7)	22.4 (17.6)	± 2.4-teaspoon change
Diet cost (dollars)	3.86 (1.92)	3.85 (1.92)	3.85 (1.92)	3.85 (1.92)	3.84 (1.92)	± 10% change

Notes: Whole and reduced-fat milk was replaced with low-fat and skim milk on a random 50–50 basis. For example, for the 15% change model, 15% of consumption reports were first randomly selected and half of those reports were replaced with low-fat milk and the other half with skim milk. Data shown correspond to survey-weighted means and SDs. *P* is from a 1-sided test, with the exception of diet cost, which is from a 2-sided test.

a Corresponds to 10% change in daily value (see Table 1 footnote for definitions).

∗ Difference between modeled diets and observed diets is significantly different (*P* < .001) from the specified benchmark value

**Table 4:** Estimated Survey-Weighted Mean (SD) Energy, Nutrient Intake, and Diet Cost After Replacing Flavored Milk^a^ Among Those Consuming Flavored Milk,^b^ 2001–2004

	Observed	Replacement With Skim and 1% White Milk on a 50:50 Basis	Benchmark
Energy, kcal	2,194 (832)	2,062 (813)∗	100 kcal
Total fat (% energy)	32.4 (5.8)	31.8 (7.2)	± 3.5% change^c^
Saturated fat (% energy)	12.0 (3.0)	11.2 (3.1)	± 1% change^c^
Cholesterol, mg	232 (172)	217 (169)	± 30-mg change^c^
Added sugar, teaspoons	24.6 (14.9)	20.0 (14.2)∗	± 2.4-teaspoon change
Calcium, mg	1,212 (566)	1,250 (566)	± 100-mg change^c^
Potassium, mg	2,654 (1,121)	2,622 (1,107)	± 350-mg change^c^
Diet cost ($)	3.90 (1.86)	3.82 (1.85)	± 10% change

Note: *P* is from a 1-sided test, with the exception of diet cost, which is from a 2-sided test.

∗ Difference between modeled diets and observed diets is significantly different (*P* < .001) from the specified benchmark value

a With skim and low-fat milk on a 50–50 basis

b n = 1,159 who consumed flavored milk

c Corresponds to 10% change in daily value (values are defined in the footnote to Table 1).

## Supplementary Data

Supplementary Tables s1 – s3
