# Supplementary material for: Potential Population-Level Nutritional Impact of Replacing Whole and Reduced-Fat Milk With Low-Fat and Skim Milk Among US Children Aged 2–19 Years
Source: J Nutr Educ Behav. 2015 Jan;47(1):61–68.e1. doi: 10.1016/j.jneb.2014.11.001 (PMC4315137; doi:10.1016/j.jneb.2014.11.001)
Supplement: Supplementary Tables s1 – s3 [file mmc1.docx]

**Supplemental Tables for Online Publication Only**

**Table S1**. Observed and modeled energy intakes after replacing whole milk, 2% milk and flavored milk with skim and low-fat milk among United States children and adolescents by age group, race/ethnicity and family income-to-poverty ratio, 2001-2004

|  | Observed | Model 1 –  Skim Milk | Model 2 –  Low-fat Milk |
| --- | --- | --- | --- |
|  |  |  |  |
| Age 2-5 |  |  |  |
| All children^a^ | 1686 (588) | 1599 (572) | 1626 (575) |
| MER consumers^b^ | 1714 (611) | 1596 (691)*** | 1633 (595) |
|  |  |  |  |
| Age 6-11 |  |  |  |
| All children^a^ | 2050 (641) | 1978 (622) | 1999 (626) |
| MER consumers^b^ | 2129 (681) | 2015 (659)** | 2049 (664) |
|  |  |  |  |
| Age 12-19 |  |  |  |
| All children^a^ | 2321 (1189) | 2274 (1169) | 2290 (1175) |
| MER consumers^b^ | 2492 (1245) | 2384 (1212) | 2420 (1222) |
|  |  |  |  |
| Non-Hispanic White |  |  |  |
| All children^a^ | 2096 (632) | 2033 (622) | 2053 (625) |
| MER consumers^b^ | 2145 (645) | 2032 (630)** | 2069 (635) |
|  |  |  |  |
| Non-Hispanic Black |  |  |  |
| All children^a^ | 2099 (1428)) | 2045 (1404) | 2061 (1411) |
| MER consumers^b^ | 2194 (1269) | 2096 (1337) | 2125 (1246) |
|  |  |  |  |
| Mexican-American |  |  |  |
| All children^a^ | 2115 (1277) | 2032 (1261) | 2056 (1264) |
| MER consumers^b^ | 2127 (1275) | 1999 (1250)*** | 2036 (1255) |
|  |  |  |  |
| <130% of IPR^c^ |  |  |  |
| All children^a^ | 2103 (1070) | 2025 (1053) | 2048 (1057) |
| MER consumers^b^ | 2123 (1006) | 2000 (979)*** | 2037 (987) |
|  |  |  |  |
| 130-349% of IPR^c^ |  |  |  |
| All children^a^ | 2055 (900) | 1995 (888) | 2015 (891) |
| MER consumers^b^ | 2111 (937) | 2006 (920) | 2040 (925) |
|  |  |  |  |
| ≥350% of IPR^c^ |  |  |  |
| All children^a^ | 2133 (765) | 2081 (754) | 2098 (757) |
| MER consumers^b^ | 2195 (789) | 2087 (770)* | 2122 (776) |
|  |  |  |  |
| Underweight (<5^th^ %ile) |  |  |  |
| All children^a^ | 2224 (1252) | 2142 (1232) | 2168 (1238) |
| MER consumers^b^ | 2272 (1272) | 2158 (1251) | 2194 (1257) |
|  |  |  |  |
| Healthy weight (5-84.9^th^ %ile) |  |  |  |
| All children^a^ | 2111 (921) | 2046 (908) | 2066 (911) |
| MER consumers^b^ | 2147 (915) | 2036 (895)** | 2071 (901) |
|  |  |  |  |
| Overweight (85-94.9^th^ %ile) |  |  |  |
| All children^a^ | 2118 (904) | 2061 (884) | 2080 (890) |
| MER consumers^b^ | 2205 (977) | 2092 (946)* | 2130 (956) |
|  |  |  |  |
| Obese (≥95^th^ %ile) |  |  |  |
| All children^a^ | 2035 (857) | 1976 (845) | 1995 (847) |
| MER consumers^b^ | 2092 (797) | 1975 (777)** | 2013 (782) |
|  |  |  |  |

^a^ All children refers to all children age 2-19 who completed a valid 24-h recall

^b^ MER (milks eligible for replacement) consumers refers to all children age 2-19 who reported consuming any type of milk eligible for replacement models, including whole fat, 2% (reduced fat) milk and flavored milk with added sugars.

^c^ IPR is income-to-poverty ratio.

Asterisks indicate whether the observed difference is greater than the specified benchmark value of 100 (* *P* < .05, ** *P* < .01, *** *P* < .001). All *P* values are one-sided.

**Table S2**. Observed and modeled percent energy from total fat after replacing whole milk, 2% milk and flavored milk with skim and low-fat milk among United States children and adolescents by age group, race/ethnicity and family income-to-poverty ratio, 2001-2004

|  | Observed | Model 1 –  Skim Milk | Model 2 –  Low-fat Milk |
| --- | --- | --- | --- |
|  |  |  |  |
| Age 2-5 |  |  |  |
| All children^a^ | 31.9 (7.1) | 28.4 (7.7) | 29.8 (7.2) |
| MER consumers^b,c^ | 32.5 (6.7) | 27.8 (7.8)*** | 29.6 (7.2) |
|  |  |  |  |
| Age 6-11 |  |  |  |
| All children^a^ | 32.5 (6.2) | 30.6 (6.7) | 31.4 (6.4) |
| MER consumers^b^ | 32.6 (6.0) | 29.5 (6.6) | 30.8 (6.3) |
|  |  |  |  |
| Age 12-19 |  |  |  |
| All children^a^ | 32.4 (9.2) | 31.1 (9.6) | 31.6 (9.3) |
| MER consumers^b^ | 33.0 (8.5) | 30.1 (9.3) | 31.3 (8.9) |
|  |  |  |  |
| Non-Hispanic White |  |  |  |
| All children^a^ | 32.2 (5.4) | 30.3 (5.7) | 31.1 (5.5) |
| MER consumers^b^ | 32.9 (5.0) | 29.5 (5.5) | 30.9 (5.2) |
|  |  |  |  |
| Non-Hispanic Black |  |  |  |
| All children^a^ | 33.4 (11.7) | 31.7 (12.7) | 32.3 (12.3) |
| MER consumers^b^ | 33.0 (10.4) | 30.0 (11.8) | 31.1 (11.2) |
|  |  |  |  |
| Mexican-American |  |  |  |
| All children^a^ | 32.4 (11.7) | 29.6 (11.4) | 30.6 (10.8) |
| MER consumers^b^ | 32.2 (9.2) | 28.4 (10.6)** | 29.8 (10.0) |
|  |  |  |  |
| <130% of IPR^c^ |  |  |  |
| All children^a^ | 32.8 (9.3) | 30.3 (10.1) | 31.3 (9.6) |
| MER consumers^b^ | 33.0 (8.5) | 29.2 (9.5)* | 30.7 (8.9) |
|  |  |  |  |
| 130-349% of IPR^c^ |  |  |  |
| All children^a^ | 32.3 (7.6) | 30.4 (8.1) | 31.2 (7.7) |
| MER consumers^b^ | 32.6 (6.9) | 29.3 (7.6) | 30.7 (7.2) |
|  |  |  |  |
| ≥350% of IPR^c^ |  |  |  |
| All children^a^ | 31.9 (6.2) | 30.5 (6.4) | 31.1 (6.2) |
| MER consumers^b^ | 32.3 (5.6) | 29.3 (7.6) | 30.6 (5.8) |
|  |  |  |  |
| Underweight (<5^th^ %ile) |  |  |  |
| All children^a^ | 33.2 (7.2) | 30.7 (7.8) | 31.7 (7.4) |
| MER consumers^b^ | 33.7 (6.6) | 30.2 (7.4) | 31.6 (7.0) |
|  |  |  |  |
| Healthy weight (5-84.9^th^ %ile) |  |  |  |
| All children^a^ | 32.3 (7.5) | 30.4 (8.0) | 31.2 (7.7) |
| MER consumers^b^ | 32.6 (6.9) | 29.2 (7.6) | 30.6 (7.2) |
|  |  |  |  |
| Overweight (85-94.9^th^ %ile) |  |  |  |
| All children^a^ | 32.2 (8.2) | 30.5 (8.6) | 31.2 (8.4) |
| MER consumers^b^ | 33.0 (7.5) | 29.6 (8.4) | 31.0 (7.9) |
|  |  |  |  |
| Obese (≥95^th^ %ile) |  |  |  |
| All children^a^ | 32.3 (8.7) | 30.4 (9.3) | 31.2 (8.9) |
| MER consumers^b^ | 32.7 (7.9) | 29.1 (9.0) | 30.6 (8.4) |
|  |  |  |  |

^a^ All children refers to all children age 2-19 who completed a valid 24-h recall

^b^ MER (milks eligible for replacement) consumers refers to all children age 2-19 who reported consuming any type of milk eligible for replacement models, including whole fat, 2% (reduced fat) milk and flavored milk with added sugars.

^c^ For children 2-3y, no more than 40% of total energy is to come from fat. For Model 1, among milk-consumers, the difference remained statistically significant (*P* < .001) after using 4% as a reference value.

^c^ IPR is income-to-poverty ratio.

Asterisks indicate whether the observed difference is greater than the specified benchmark value of 3.5% (* *P* < .05, ** *P* < .01, *** *P* < .001). All *P* values are one-sided.

**Table S3**. Observed and modeled percent energy from saturated fat after replacing whole milk, 2% milk and flavored milk with skim and low-fat milk among United States children and adolescents by age group, race/ethnicity and family income-to-poverty ratio, 2001-2004

|  | Observed | Model 1 –  Skim Milk | Model 2 –  Low-fat Milk |
| --- | --- | --- | --- |
|  |  |  |  |
| Age 2-5 |  |  |  |
| All children^a^ | 11.9 (3.4) | 9.5 (3.3)*** | 10.5 (3.1)*** |
| MER consumers^b^ | 12.6 (3.3) | 9.3 (3.4)*** | 10.6 (3.6)*** |
|  |  |  |  |
| Age 6-11 |  |  |  |
| All children^a^ | 11.5 (2.9) | 10.1 (2.9)*** | 10.7 (2.8) |
| MER consumers^b^ | 12.0 (2.7) | 9.7 (2.8)*** | 10.7 (2.7)*** |
|  |  |  |  |
| Age 12-19 |  |  |  |
| All children^a^ | 11.1 (3.9) | 10.2 (3.8) | 10.6 (3.8) |
| MER consumers^b^ | 12.0 (3.6) | 9.8 (2.8)*** | 10.8 (3.6)*** |
|  |  |  |  |
| Non-Hispanic White |  |  |  |
| All children^a^ | 11.5 (2.5) | 10.2 (2.4)*** | 10.8 (2.3) |
| MER consumers^b^ | 12.4 (2.3) | 9.9 (2.3)*** | 11.0 (2.2)*** |
|  |  |  |  |
| Non-Hispanic Black |  |  |  |
| All children^a^ | 11.3 (4.9) | 10.1 (5.0)*** | 10.6 (4.9) |
| MER consumers^b^ | 11.7 (4.3) | 9.6 (4.6)*** | 10.4 (4.4)*** |
|  |  |  |  |
| Mexican-American |  |  |  |
| All children^a^ | 11.4 (4.6) | 9.6 (4.7)*** | 10.3 (4.5) |
| MER consumers^b^ | 11.9 (4.1) | 9.1 (4.4)*** | 10.3 (4.1)*** |
|  |  |  |  |
| <130% of IPR^c^ |  |  |  |
| All children^a^ | 11.6 (4.1) | 9.9 (4.1)*** | 10.6 (3.9) |
| MER consumers^b^ | 12.3 (3.7) | 9.5 (3.9)*** | 10.7 (3.6)*** |
|  |  |  |  |
| 130-349% of IPR^c^ |  |  |  |
| All children^a^ | 11.5 (3.4) | 10.1 (3.4)*** | 10.7 (3.2) |
| MER consumers^b^ | 12.2 (3.1) | 9.7 (3.2)*** | 10.8 (3.1)*** |
|  |  |  |  |
| ≥350% of IPR^c^ |  |  |  |
| All children^a^ | 11.2 (2.8) | 10.1 (3.9) | 10.6 (3.9) |
| MER consumers^b^ | 11.8 (2.6) | 9.6 (2.7)*** | 10.6 (2.5)*** |
|  |  |  |  |
| Underweight (<5^th^ %ile) |  |  |  |
| All children^a^ | 12.0 (3.3) | 10.2 (3.3)*** | 10.9 (3.2) |
| MER consumers^b^ | 12.5 (3) | 10.0 (3.1)*** | 11.1 (2.9)** |
|  |  |  |  |
| Healthy weight (5-84.9^th^ %ile) |  |  |  |
| All children^a^ | 11.4 (3.4) | 10.0 (3.4)*** | 10.6 (3.3) |
| MER consumers^b^ | 12.0 (3.1) | 9.6 (3.2)*** | 10.7 (3.1)*** |
|  |  |  |  |
| Overweight (85-94.9^th^ %ile) |  |  |  |
| All children^a^ | 11.2 (3.5) | 10.0 (3.5)** | 10.5 (3.4) |
| MER consumers^b^ | 12.1 (3.3) | 9.7 (3.5)*** | 10.7 (3.3)*** |
|  |  |  |  |
| Obese (≥95^th^ %ile) |  |  |  |
| All children^a^ | 11.3 (3.7) | 9.9 (3.7)** | 10.5 (3.5) |
| MER consumers^b^ | 12.2 (3.5) | 9.6 (3.6)*** | 10.7 (3.4)*** |
|  |  |  |  |

^a^ All children refers to all children age 2-19 who completed a valid 24-h recall

^b^ MER (milks eligible for replacement) consumers refers to all children age 2-19 who reported consuming any type of milk eligible for replacement models, including whole fat, 2% (reduced fat) milk and flavored milk with added sugars.

^c^ IPR is income-to-poverty ratio.

Asterisks indicate whether the observed difference is greater than the specified benchmark value of 1% (* *P* < .05, ** *P* < .01, *** *P* < .001). All *P* values are one-sided.
